# Protection of Retina by Mini-αA in NaIO_3_-Induced Retinal Pigment Epithelium Degeneration Mice

**DOI:** 10.3390/ijms16011644

**Published:** 2015-01-12

**Authors:** Jinglin Zhang, Xiujuan Zhao, Yu Cai, Yonghao Li, Xiling Yu, Lin Lu

**Affiliations:** State Key Laboratory of Ophthalmology, Zhongshan Ophthalmic Center, Sun Yat-sen University, Guangzhou 510060, China; E-Mails: zhjinglin@126.com (J.Z.); zhxj77retina@163.com (X.Z.); caiyu672@163.com (Y.C.); liyhao@mail.sysu.edu.cn (Y.L.); yuxiling246@163.com (X.Y.)

**Keywords:** mini-α, NaIO_3_-induced retinal pigment epithelium, cell apoptosis

## Abstract

Background: Studies have shown that mini-αA can protect retinal pigment epithelium (RPE) cells from apoptosis. However, no *in vivo* study concerning the anti-apoptotic function of mini-αA has been conducted yet. Methods: MTT assay, HE staining and TUNEL assay were used to assess levels of cells, and an animal model was established to examine the protective effects of mini-αA against NaIO_3_-induced RPE cell apoptosis. Western blot analysis and RT-qPCR were performed to explore the possible mechanism of mini-αA’s protective function against NaIO_3_-induced RPE cell apoptosis. Results: Results from *in vivo* and animal experiments showed that mini-αA antagonized NaIO_3_-induced RPE cell apoptosis. Further investigation into how mini-αA provided protection against NaIO_3_-induced RPE cell apoptosis showed that mini-αA reduced NaIO_3_-induced RPE cell apoptosis and autophagy. In addition, unfolded protein response was also involved in the protective effects of mini-αA against NaIO_3_-induced RPE cell apoptosis. Conclusions: mini-αA can antagonize RPE cell apoptosis induced by NaIO_3_. A possible mechanism is by inhibition of apoptosis by repressing autophagy and endoplasmic reticulum stress.

## 1. Introduction

Crystallins are classified into three main types: α, β and γ, among which α-crystallin has the highest quantity in the crystalline lens; α-crystallin is a member of the small heat shock proteins (HSPs) family [1]. Its multifunctional properties have earned it lots of attention. With its chaperone-like properties, α-crystallin can maintain its protein conformation when under stress, and plays a critical role in coping with internal and external stress α-crystallin comprises two types of related subunits: αA-crystallin and αB-crystallin. αA-crystallins are located mainly in the crystalline lens, although a small amount is expressed in the spleen and the thymus [2]. Studies showed that a marked increase of mRNA and expression of αA-crystallin and αB-crystallin was observed in models of light-induced injury, retinal trauma [3,4,5] and other acute retinal degeneration, suggesting α-crystallin plays an important part in the early stage of retinal degeneration. Peng Zhou, *et al.* [6] conducted a meta-analysis concerning the association between Geographic Atrophy (GA) and cataract or cataract surgery, and found that cataracts are associated with an increased risk of developing GA. In the mouse model of NaIO_3_-induced GA, increased expression of αA-crystallin mRNA and αA-crystallin was observed. NaIO_3_-induced GA was more severe in αA-crystallin knockout mice. These all suggest that αA-crystallin can inhibit GA induced by NaIO_3_.

In a study conducted by N. Pasupuleti, *et al.* [7] found that there is a direct correlation between the anti-apoptotic function of αA-crystallin and its chaperone activity. Mini-αA, a 19 amino acid peptide sequence (DFVIFLDVKHFSPEDLTVK), is the functional site of αA-crystallin and possesses chaperon-like activity [8]. Previous *in vitro* studies have shown that mini-αA can inhibit the activation of caspase-3, thus protecting RPE cells from apoptosis induced by hydrogen peroxide [9]. However, no *in vivo* study concerning the anti-apoptotic function of mini-αA has been performed. This study investigates the effects of mini-αA on mice with NaIO_3_-induced retinal degeneration.

## 2. Results

### 2.1. Mini-αA Protected ARPE-19 from NaIO_3_-Induced Apoptosis

**Figure 1:**
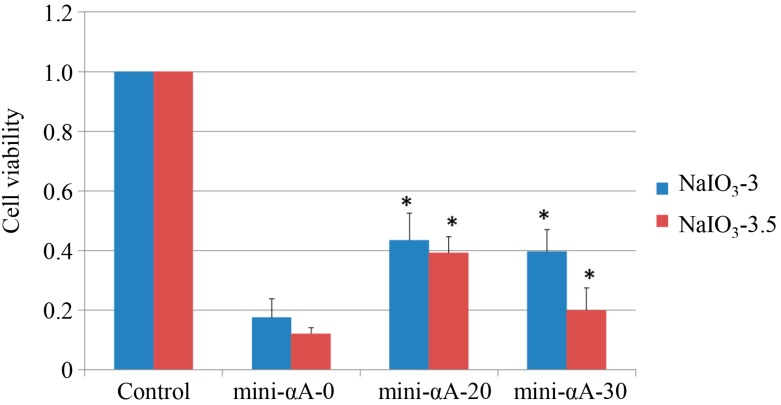
Cell viability assessed by MTT assay. Induction of NaIO_3_ at the concentration of 3 and 3.5 mM can both reduce the viability of RPE cells. Mini-αA at the concentrations of 20 and 30 μM can both inhibit the reduction of cell viability caused by NaIO_3_, with the best result achieved by mini-αA at the concentration of 20 μM. Control: normal ARPE-19 cells; mini-Αa-0: no mini-αA treatment before NaIO_3_ induction; mini-Αa-20: treated with mini-αA at the concentration of 20 μM; mini-Αa-30: treated with mini-αA at the concentration of 30 μM. * *p* < 0.05 *vs.* mini-Αa-0.

To investigate the protective effects of mini-αA against NaIO_3_-induced retinal degeneration via an *in vitro* test, an MTT assay was performed to assess viability of ARPE-19 cells. ARPE-19 cells were treated with NaIO_3_ (concentration: 3 and 3.5 mM) 24 h after mini-αA treatment (0, 20, 30 µM, respectively). MTT assay was performed on the cells 36 h later. We found that, compared with that of normal ARPE-19 cells, the viability of cells treated with NaIO_3_ is much lower. The mini-αA used (both 20 and 30 μM) can inhibit the reduction of cell viability caused by NaIO_3_, with cells treated with 20 μM mini-αA showing the greatest survival. The difference between cells treated with and without mini-αA is statistically significant (Figure 1).

### 2.2. Mini-αA Reduced Retinal Damage in Mice with NaIO_3_-Induced Retinal Degeneration

**Figure 2:**
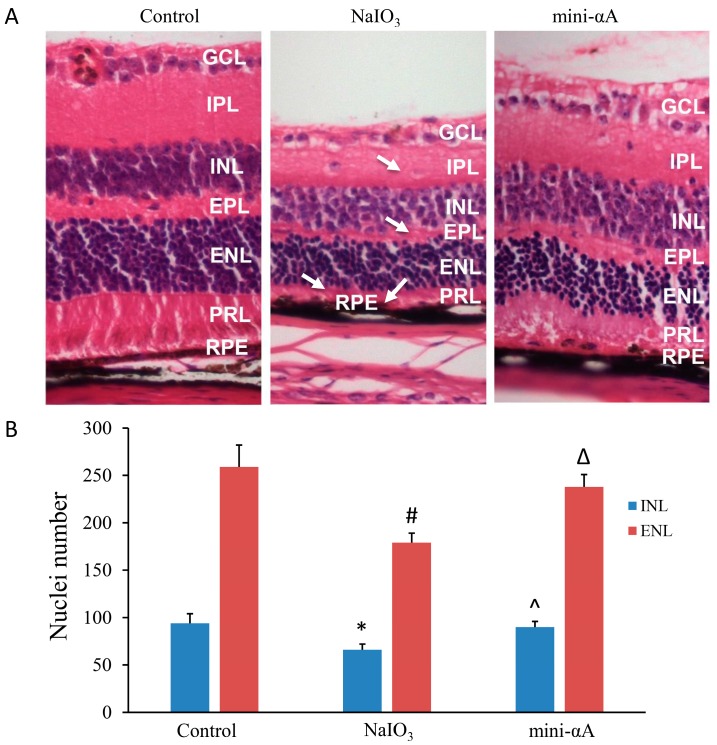
mini-αA treatment protect the injuries induced by NaIO_3_. (**A**) HE staining of retina two weeks after mouse model of NaIO_3_-induced retinal degeneration was established. (Control) Retina of normal control group, whose layers were organized and cellular stratification neat; (NaIO_3_) Retina of NaIO_3_ group; (mini-αA group) Retina of mini-αA group. GCL: ganglion cell layer, IPL: internal plexiform layer, INL: internal nuclear layer, EPL: external plexiform layer, ENL: external nuclear layer, PRL: photoreceptor layer, RPE: retinal pigmented epithelium. Arrows indicated the retina damages caused by NaIO_3._ Thickness reduction was seen in GCL, EPL, PRL. RPE layers were discontinuous. Magnification: 400×; (**B**) Nuclei number of INL and ENL was counted. Significant reduction of nuclei number of INL and ENL was observed in NaIO_3_ group compared to the control and the mini-αA group mini-αA treatment reversed the nuclei number reduction. Quantification of nuclei number was performed with six independent experiments. * *p* < 0.05 *vs.* INL of the control; # *p* < 0.05 *vs.* ENL of the control; ^ *p* < 0.05 *vs.* INL of the NaIO_3_ group; Δ *p* < 0.05 *vs.* ENL of the NaIO_3_ group.

To further validate that mini-αA provides protection against NaIO_3_-induced retinal degeneration, this study established a mouse model of NaIO_3_-induced retinal degeneration. HE staining was performed on retinal Sections two weeks after the model was successfully established. Results showed that, compared with those of the normal mice, the retinas of the mice with NaIO_3_-induced retinal degeneration were obviously thinner, with thinning of the photoreceptor layer being most evident, and the layers of the retina were disorganized with displaced cells. The structure of the RPE layer was not clear, and almost no RPE cells were noted. The retinas of mini-αA group were slightly thinner than those of normal mice, but thicker than retinas induced by NaIO_3_, and the cellular stratification of the retinas of mini-αA group was neater. Similar to retinas induced by NaIO_3_, a reduction in the thickness of the photoreceptor layer was most evident, the structure of RPE layer unclear, and almost no RPE cells were identified (Figure 2A). Quantification of nuclei of the internal nuclear layer (INL) and external plexiform layer (ENL) confirmed the protection of retinas damage caused by NaIO_3_ (Figure 2B). These results suggest that, in agreement with the *in vitro* experiment, mini-αA can protect retinas against injuries caused by NaIO_3_.

**Figure 3:**
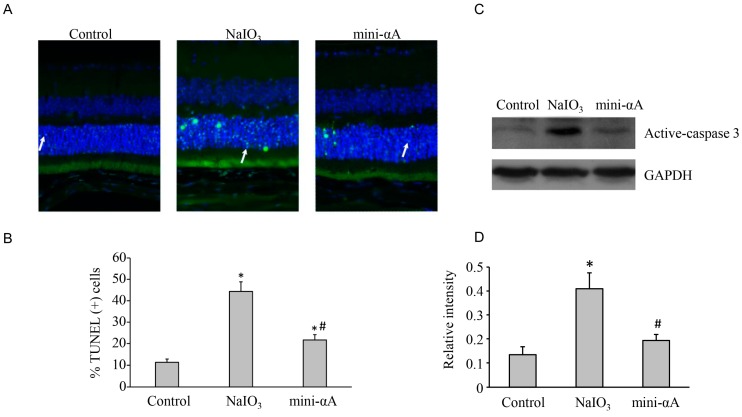
TUNEL assay was performed 3 days after the establishment of the mouse model of NaIO_3_-induced retinal degeneration. (**A**) Almost no TUNEL-positive cells were detected in normal control group; a large number of TUNEL-positive cells were found in the external nuclear layer in the retinas of NaIO_3_ group (white arrow); several TUNEL-positive cells were observed in the external nuclear layer in mini-αA group (white arrow), but much less than those observed in NaIO_3_ group. Magnification: 400×; (**B**) Quantification of dead cells by TUNEL assay from at least five independent experiments. TUNEL-positive cells were counted and data were expressed as percent of total death cells; (**C**) Western blot analysis of caspase expression levels in each subgroup; (**D**) Quantification of band intensity of caspase from three independent experiments on (**C**). * *p* < 0.05 *vs.* Control; # *p* <0.05 *vs.* NaIO_3_.

Previous studies suggest that the reason why NaIO_3_ can cause thinning of the retina is that RPE cell apoptosis leads to photoreceptor apoptosis. Three days after successful establishment of the model, a TUNEL assay was performed to assess cell apoptosis in a control group, NaIO_3_ group and mini-αA group. Results showed that, although there was no significant change in retinal thickness and cellular stratification in each group, only a few TUNEL-positive cells were observed in the retinas of the control group, whereas a large number of TUNEL-positive cells were found in both the external and internal nuclear layers in the retinas of the NaIO_3_ group. TUNEL-positive cells detected in both the external and internal nuclear layers in the retina of mini-αA group were less than those detected in NaIO_3_ group, but more than those found in the control group. This explains why the retinas of mini-αA group were thicker than those of NaIO_3_ group but thinner than those of normal control group in Week two (Figure 3).

### 2.3. Mini-αA Reduced NaIO_3_-Induced Apoptosis and Autophagy Level

**Figure 4:**
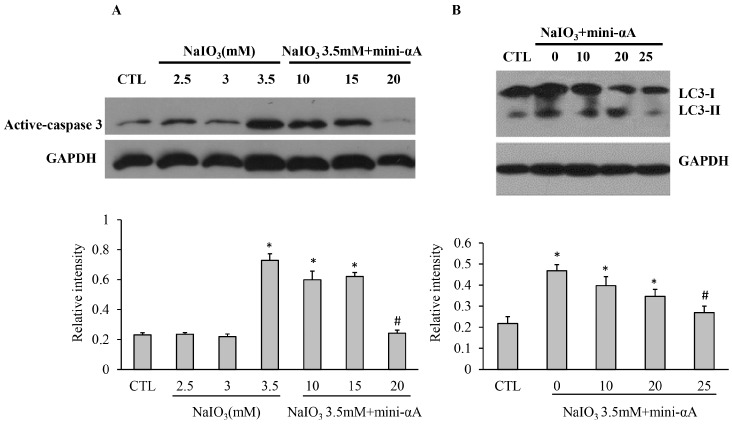
Western blotting was performed to detect caspase and 3LC3- I/II protein expression levels of each subgroup. (**A**) Caspase expression levels of each subgroup and quantification of the band intensity of caspase from there independent experiments; (**B**) 3LC3- I/II protein expression levels in each group and quantification of the band intensity from there independent experiments. GAPDH was used as the internal control. CTL: normal ARPE-19 cells; NaIO_3_: cells induced with NaIO_3_; NaIO_3_ + mini-αA: cells treated first with mini-αA and then with NaIO_3_. * *p* < 0.05 *vs.* Control; # *p* < 0.05 *vs.* NaIO_3_.

To explore the mechanism of mini-αA’s protective function against NaIO_3_-induced retinal degeneration, ARPE-19 cells were induced by NaIO_3_ at 2.5, 3 and 3.5 mM. Meanwhile, three subgroups treated with NaIO_3_ at 3.5 mM were set up, and were treated with mini-αA at concentrations of 10, 15 or 20 μM. Western blot analysis revealed that the expression of active-Caspase3 in cells treated with NaIO_3_ at 3.5 mM was much higher than that in normal cells, and the expression in cells treated with 3.5 mM NaIO_3_ and 20 μmM mini-αA decreased dramatically (Figure 4A).

In addition, to examine effects of mini-αA on autophagy, Western blot analyses were performed to detect the expression level of LC3-I/II in cells treated with 3.5 mM NaIO_3_. It was found that the level of LC3-II protein was relatively low in normal RPE cells, but evidently elevated in NaIO_3_-treated cells. Cells treated with 3.5 mM NaIO_3_ were treated with mini-αA at 10, 15 or 20 μM. The expression level of LC3-II protein was evidently decreased in cells treated with 20 μM mini-αA, and thus showed no significant difference with the normal RPE group (Figure 4B). These results showed that NaIO_3_ induced not only RPE cell apoptosis, but also autophagy of RPE cells. Additionally, treatment with mini-αA at a concentration of 20 μM can reduce NaIO_3_-induced apoptosis and autophagy levels.

### 2.4. Mini-αA’s Protective Function against NaIO_3_-Induced RPE Cell Apoptosis Is Related with UPR

**Figure 5:**
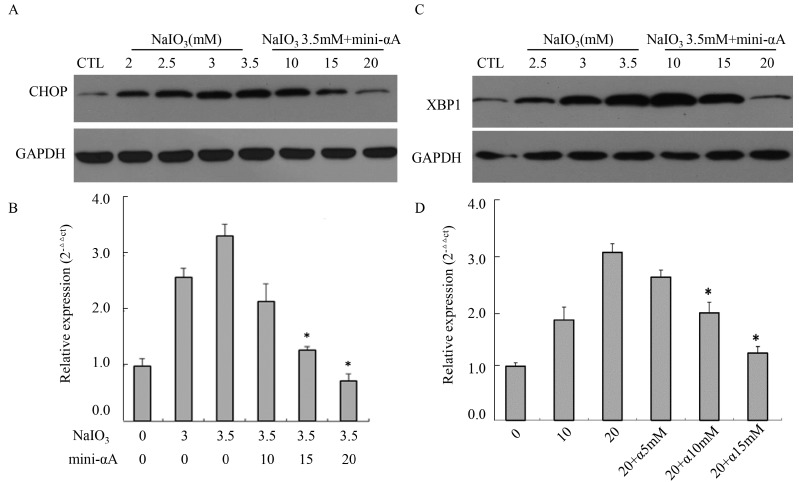
Effects of mini-αA on CHOP and XBP1 expression level. (**A**) Western blot analysis was performed to detect the expression level of CHOP protein in each group; (**B**) RT-qPCR was used to detect the expression level of *CHOP* mRNA in each group. CTL: normal ARPE-19 cells; * *p* < 0.05; (**C**) Western blot was performed to detect XBP1 protein expression level in each group; (**D**) RT-qPCR was used to detect the expression level of *XBP1* mRNA in each group. NaIO_3_ induction elevated the expression level of XBP1 in RPE cells, while mini-αA treatment inhibited the increase caused by NaIO_3_ induction. CTL: normal ARPE-19 cells, NaIO_3_: cells induced with NaIO_3_; NaIO_3_ + mini-αA: cells treated first with mini-αA and then with NaIO_3_. GAPDH was used as the internal. * *p* < 0.05.

To examine the relationship between unfolded protein response (UPR) and mini-αA’s protective function against NaIO_3_-induced RPE cell apoptosis, Western blot analysis was performed to detect CHOP expression levels in cells induced by NaIO_3_ at concentrations of 2, 2.5, 3 and 3.5 mM. We discovered that, compared with normal untreated RPE cells, there was a marked increase in CHOP protein expression in cells treated with NaIO_3_, and the expression level rose as the concentration of NaIO_3_ rose. After being treated with mini-αA at 10, 15 or 20 μM, CHOP protein expression levels of RPE cells induced by 3.5 mM NaIO_3_ were evidently repressed, with 20 μM mini-αA showing the best inhibitive effect (Figure 5A). In addition, RT-qPCR was used to detect expression levels of *CHOP* mRNA in each group. The result was the same as that of the Western blot analysis. The *CHOP* mRNA expression levels in cells induced by NaIO_3_ at 2, 2.5, 3 or 3.5 mM were much higher than that of normal cells, and the mRNA expression level rose as the concentration of NaIO_3_ rose. After mini-αA treatment, the mRNA expression level decreased, with 20 μM mini-αA showing the greatest inhibition (Figure 5B).

According to the results of Western blot analysis, the level of XBP1 increased remarkably after NaIO_3_ induction, but this increase was inhibited in a dose-dependent manner after mini-αA treatment (Figure 5C). Meanwhile, RT-qPCR was used to detect the expression level of *XBP1* mRNA in each group, and the results coincided with those of the Western blot analysis (Figure 5D). These results suggest that mini-αA’s protective function against NaIO_3_-induced RPE cell apoptosis is related to UPR.

## 3. Discussion

In our study, results from the *in vitro* MTT cell viability assay revealed that mini-αA protected RPE cells in a dose-dependent manner. Within a certain dose range, the protective effects of mini-αA increased with its concentrations. This coincides with previous studies [10].

To validate that mini-αA protects RPE cells against NaIO_3_-induced programmed cell death, we established a mouse model of NaIO_3_-induced retinal degeneration. Our results showed that retinal damage caused by NaIO_3_ was reduced when the H&E-stained retinal sections were examined 14 days after mini-αA administration (10 μL, 500 μM). NaIO_3_-induced retinal degeneration models are created by selectively causing RPE injury, leading to injuries of photoreceptors and other tissues [11]. NaIO_3_ is an inorganic oxidizing agent and the severity of retinal injury is proportional to the dose of NaIO_3_ [12]. With doses as high as 40–100 mg/kg, only a few [13] or even no [14,15,16] RPE cells were regenerated, while when low dose of NaIO_3_ was administered (15–25 mg/kg), the RPE layer was partially restored and its functions recovered to certain extent after injury. Furthermore, the recovery of this function developed from peripheral retina to central retina [17]. Studies showed that cell swelling was observed in outer segment (OS) layer of photoreceptors as early as 1 h post NaIO_3_ injection [18]. As time went by, the injury deteriorated and reached its peak 7–14 days post injection [11,15,19]. Therefore, in this study we chose to use a high dose of NaIO_3_ (40 mg/kg) to establish the model, and to subject central retina to HE stain 14 days post model establishment.

Some studies have shown that photoreceptor apoptosis reaches its peak three days after injection, regardless of NaIO_3_ dosage (high or low) or the location of photoreceptors (central retina or peripheral retina) [11,12]. Therefore, we chose to perform TUNEL assay on retina Sections three days after establishment of our model. Our TUNEL assay results suggest that mini-αA can protect RPE cells against NaIO_3_ damage, reducing cell death. We found that three days after NaIO_3_ injection (40 mg/kg), the RPE layer had been damaged almost completely with only a few cells remaining, which coincides with previous reports [12]. Only a layer of residual melanin alongside Bruch’s membrane was visible. Our study also found that mini-αA can inhibit photoreceptor apoptosis. However, because the RPE layer was also damaged, the retinas of mini-αA group were thinner than normal retinas on day 14, though thicker than those of the control group.

Studies have shown that endoplasmic reticulum stress (ERS) and UPR play critical roles in certain diseases related to photoreceptor apoptosis, suggesting that therapeutic approaches targeting these pathways may be effective [20,21,22]. In our study, after treating RPE cells with NaIO_3_, the expression levels of CHOP and XBP1 proteins and their mRNA all showed a marked increase. After mini-αA was administered, however, the expression levels of these ERS-associated proteins and mRNA decreased, suggesting that the anti-apoptotic function of mini-αA is associated with ERS. Notably, the expression level of LC3II, a marker for autophagy, in NaIO_3_ treated RPE cells was elevated remarkably. Meanwhile, that mini-αA can inhibit autophagy suggests that autophagy is involved in the anti-apoptotic process of mini-αA. As the endoplasmic reticulum can serve as a membrane source and build structures for autophagy, its dysfunction can not only affect the process of autophagy, but also trigger autophagy and degrade abnormal endoplasmic reticulum. This process is called ER-phagy [23]. When the endoplasmic reticulum malfunctions, abnormal proteins will accumulate in endoplasmic reticulum, activating UPR. This response includes three major pathways: PERK, ATF6 and IRE1 [24,25,26]. PERK and ATF6 both can trigger autophagy [27]. The results of our study suggest that inhibiting apoptosis, autophagy and ERS all contribute to the anti-apoptotic function of mini-alpha A-crystallin, though their correlation is not yet clear.

In addition, studies show that RPE cell death after exposure to NaIO_3_ involved not only apoptosis, but also necrosis [11]. As a member of the HSP family, anti-inflammation is one of the significant biological functions of αA-crystallin. The protective effects of αA-crystallin in photoreceptors in experimental uveitis were considered to be associated with its anti-inflammatory function [28,29,30]. Therefore, the protective effects of mini-αA against NaIO_3_-induced retinal degeneration may be partly associated with its anti-inflammatory function.

## 4. Material and Methods

### 4.1. Cell Culture

The human RPE cell line ARPE-19 was purchased from American Type Culture Collection (ATCC) (Manassas, VA, USA). The cells were cultured in Dulbecco’s Modified Eagle’s Medium (DMEM) containing high glucose (4.5 mg/mL), l-glutamine and sodium pyruvate (PAA, Coelbe, Germany). The medium was supplemented with penicillin/streptomycin and 10% porcine serum (PAA). The cells were incubated at 37 °C in a 5% CO_2_ atmosphere.

The culture medium was removed using a dropper, and the cells were washed twice with PBS. For a model of NaIO_3_-induced RPE cell apoptosis, the cells were plated in Dulbecco’s Modified Eagle Medium: Nutrient Mixture F-12 (DMEM/F12) with different concentrations of NaIO_3_ (Sigma, St. Louis, MO, USA), and incubated for 24 h in a carbon dioxide incubator. For a model of mini-αA protecting RPE cells against NaIO_3_-induced apoptosis, the cells were maintained in DMEM/F12 with different concentrations of mini-αA (donated by Dr. Sharma from Department of Ophthalmology, University of Missouri-Columbia), and incubated for 4 h in a carbon dioxide incubator. The culture medium was removed; the cells were washed with PBS, immersed in DMEM/F12 containing NaIO_3_ and incubated for 24 h in a CO_2_ incubator.

### 4.2. Animals

Male C57BL mice (6 to 8 weeks old, 18 to 22 g) were purchased from Southern Medical University Laboratory Animal Center (Guangzhou, China). All animal procedures were performed in compliance with the approved protocols of Sun Yat-sen University and ethical approval for this study was given by the Institute Research Medical Ethics Committee of Sun Yat-sen University. The mice were housed and fed in an air-conditioned room on a 12-h light/dark cycle in a standard laboratory. The mice were divided into three groups (*n* = 6 mice per group): (1) control group: no treatment was given; (2) NaIO_3_ group: normal saline was injected intravitreally, and cells were treated with NaIO_3_; (3) mini-αA group: αA-crystallin, and cells were treated with NaIO_3_.

To establish an animal model of NaIO_3_-induced retinal degeneration, the mice were injected intraperitoneally with 4.3% chloral hydrate (430 mg/kg, Pharmacy Department, Zhongshan Ophthalmic Centre, Sun Yat-sen University) prior to intravitreal injection of mini-αA. After anesthesia, compound tropicamide eye drops (Shenyang Xingqi Pharmaceutical Co., Ltd., Shenyang, China) were injected, and lidocaine eye drops (Pharmacy Department, Zhongshan Ophthalmic Centre, Sun Yat*-*sen University) were administered 10 min later when the pupils of the mice dilated. With the aid of a surgical microscope, the needle of a microsyringe was inserted 0.5 mm posterior to the corneoscleral limbus into the vitreous cavity, avoiding the crystalline lens, and 10 μL of mini-αA solution (500 μM) was quickly pushed in. Mice in the NaIO_3_ group were injected with 10 μL of normal saline. Following injection, neomycin eye drops (Pharmacy Department, Zhongshan Ophthalmic Centre, Sun Yat-sen University) were administered. Mice with complications, such as intraocular hemorrhage, retinal detachment or lens damage were excluded from the experiment. The mice were injected with NaIO_3_ (5 mg/mL, 40 mg/kg) slowly via the dorsal vein of the penis [31].

### 4.3. Tissue Processing

Mice were euthanatized three or fourteen days after mini-αA administration by cervical dislocation. After the eyes were obtained, they were placed in 40% paraformaldehyde at room temperature for 24 h, dehydrated with a series EtOH gradient, cleared in xylene and embedded in paraffin. Specimens of 5 μm thickness were sectioned parasagittally and counterstained with hematoxylin-eosin. Three hundred micro meters away from optic disc is the central retina, and this structure was carefully studied [12].

### 4.4. TUNEL Assay

The slides were washed with fresh xylene twice (5–10 min each time), rehydrated in a graded series of ethanol (absolute ethanol for 5 min, 90%, 70% and 50% ethanol each for 2 min) and rinsed in PBS for 2 min. They were then incubated in proteinase K (without DNase, 20 μg/mL, 10 mM Tris, pH 7.4) for 15 min at room temperature and rinsed again three times in PBS. Instructions for the TUNEL *In situ* Cell Death Detection Kit (Roche, Indianapolis, IN, USA) were followed. The slides were counterstained with 5 µg/mL Hoechst, incubated for another 15 min, rinsed in PBS three more times, and then sealed. Finally, a fluorescence microscope was used to study the specimens.

### 4.5. MTT Assay

ARPE-19 cells were seeded into a 96 well-plate, with 10^3^–10^4^ cells in each well, and incubated at 37 °C in a 5% CO_2_ incubator. When the cells grew to 90% confluence, 200 μL medication (NaIO_3_, mini-αA) was added to the different groups according to the study design. Six wells were used for each of the three groups, and the cells were then returned to the incubator. After 36 h, MTT solution (5 mg/mL, 50 μL/well) was added and the cells were incubated for another 4 h. The culture medium was aspirated and 150 μL/well DMSO was added. The microtiter plate was placed on a microplate shaker for 15 min. Absorbance was measured for each well using a microplate reader at 490 nm.

### 4.6. RT-qPCR

Total cell RNA was isolated using a TRIzol reagent (Invitrogen, Carlsbad, CA, USA). The first strand of cDNA was synthesized with 1 mg of total RNA, oligo (dT) primer and AMV reverse transcriptase (Promega, Madison, WI, USA). Table 1 provides the sequences for the primers used in RT-PCR. Dissociation/melting-curve analysis and 1% agarose gel electrophoresis were performed to check the specificity of the PCR amplification products. Quantification analysis of *CHOP* and *XBP1* mRNA was normalized with GAPDH serving as the internal control.

**Table 1:** RT-qPCR Primers.

Primer	Sequence (5'–3')
*CHOP-F*	GGGAGCTGGAAGCCTGGTATG
*CHOP-R*	GACCTCTGCTGGTTCTGGCTC
*XBP1-F*	GACACGCTTGGGGATGAATGC
*XBP1-R*	TGTTCTGGGGAGGTGACAAC
*GAPDH-F*	AACGGATTTGGTCGTATTGG
*GAPDH-R*	TGGAAGATGGTGATGGGATT

### 4.7. Western Blot

Proteins were separated and transferred to PVDF membranes. The membranes were incubated overnight at 4 °C with the following monoclonal antibodies: rabbit anti-human cleaved caspase-3 (1:1000), mouse anti-human CHOP (1:500), rabbit anti-human XBP1 (1:500), rabbit anti-human GAPDH (1:10,000). Thereafter, the membranes were incubated with HRP-labeled anti-rabbit/anti-mouse secondary antibodies (1:10,000) for 1 h at room temperature. The blots were exposed to X-rayed in a dark room and the films were developed.

## 5. Conclusions

This study demonstrated mini-αA can repress NaIO_3_-induced RPE cell apoptosis. Inhibition of apoptosis by repressing autophagy and endoplasmic reticulum stress is a possible mechanism of this protective function. That mini-αA protects mice from NaIO_3_-induced retinal degeneration suggests mini-αA may be of great value in clinical applications.
